# Chromosome Evolution in Connection with Repetitive Sequences and Epigenetics in Plants

**DOI:** 10.3390/genes8100290

**Published:** 2017-10-24

**Authors:** Shu-Fen Li, Ting Su, Guang-Qian Cheng, Bing-Xiao Wang, Xu Li, Chuan-Liang Deng, Wu-Jun Gao

**Affiliations:** College of Life Sciences, Henan Normal University, Xinxiang 453007, China; lishufen83@163.com (S.-F.L.); suting534@163.com (T.S.); chengguangqian1124@163.com (G.-Q.C.); wangbingxiao39@163.com (B.-X.W.); lixu19921223@163.com (X.L.); dcl75@163.com (C.-L.D.)

**Keywords:** plant chromosome evolution, repetitive sequences, transposable elements, epigenetic modification

## Abstract

Chromosome evolution is a fundamental aspect of evolutionary biology. The evolution of chromosome size, structure and shape, number, and the change in DNA composition suggest the high plasticity of nuclear genomes at the chromosomal level. Repetitive DNA sequences, which represent a conspicuous fraction of every eukaryotic genome, particularly in plants, are found to be tightly linked with plant chromosome evolution. Different classes of repetitive sequences have distinct distribution patterns on the chromosomes. Mounting evidence shows that repetitive sequences may play multiple generative roles in shaping the chromosome karyotypes in plants. Furthermore, recent development in our understanding of the repetitive sequences and plant chromosome evolution has elucidated the involvement of a spectrum of epigenetic modification. In this review, we focused on the recent evidence relating to the distribution pattern of repetitive sequences in plant chromosomes and highlighted their potential relevance to chromosome evolution in plants. We also discussed the possible connections between evolution and epigenetic alterations in chromosome structure and repatterning, such as heterochromatin formation, centromere function, and epigenetic-associated transposable element inactivation.

## 1. Introduction

Chromosomes are the main carriers of genetic material within the nuclei of all eukaryotic cells. A molecule of linear DNA, and the associated proteins bound together, form the substance foundation of chromosomes [1]. Each chromosome morphologically contains chromosome arm(s), centromere, telomere, euchromatin, and heterochromatin [2]. As the unit of inheritance, chromosomes have evolved continuously with the evolution of organisms. The distinct features of chromosomes all undergo evolutionary changes. Thus, the specific chromosome complement of a eukaryotic organism (i.e., the karyotype) may vary considerably among groups of eukaryotes [3,4]. The patterns of chromosomal evolution exhibit extensive diversity, which may be influenced by many causal factors [5]. However, it is generally believed that the karyotype varies extensively in different species owing to the acquisition, deletion, modification, and rearrangement of nuclear DNA [6,7]. 

The eukaryote genome, i.e., the DNA content of chromosomes, is replete with different classes of repetitive DNA sequences, including transposable elements (TEs) and tandem repeats (satellites, minisatellites, and microsatellites) [8]. The repetitive sequences are particularly abundant in plant genomes, for example, 85 to 90% of the maize genome and the wheat genome are occupied by TEs [9,10,11]. Growing evidence suggests that a large fraction of repetitive sequences can have tremendous effects on the function and evolution of genomes and chromosomes [12,13,14,15]. Recent available high-throughput sequence data coupled with high-resolution cytological techniques have greatly informed our understanding of the contribution of highly repeated sequences to chromosome evolution in plants. In addition, epigenetic modification, an inseparable partner of repetitive sequences, plays important roles in many aspects of plants, including chromosome evolution. In this study, we investigated recent advances in relation to repetitive sequences and chromosome evolution in plants, focusing on the roles of repetitive sequences in the various events of chromosome evolution in plants. The possible functional roles of epigenetic modification in plant chromosome evolution were also discussed.

## 2. Chromosome Evolution in Plants

With the continuous evolution of plant species, the karyotypes of lineages are under evolutionary pressure and the chromosomes evolve through a combination of processes. Thus, the number, size, composition, structure, and shape of chromosomes differ among groups of plants, even those within a closely related taxa [3].

In accordance with the high plasticity of the nuclear genome sizes of plants [16], the size of linear metaphase chromosomes varies from less than 1 µm to more than a few tens of microns in the plant kingdom. The total size of metaphase chromosomes is generally determined by the genome size, that is, a large genome size indicates a huge total size of metaphase chromosomes [3]. The shape of monocentric chromosomes is determined by the position of the centromere, the presence of nucleolus organizing regions (NOR), and the position of NOR. The centromere, also called primary constriction, can subdivide a chromosome into “arms”, whereas the arm ratio may vary based on the position of the centromere. NOR generates a secondary constriction and a distal “satellite” in a given chromosome. The number of chromosomes can also display significant diversity, with somatic chromosome numbers from four (*n* = 2) in *Haplopappus gracilis* [17] to approximately 1440 (*n* = 720) in the fern *Ophioglossum reticulatum* [18]. 

The main factors that influence plant chromosome evolution are chromosome rearrangement and polyploidy [19,20,21,22]. The breakage and subsequent ligation of chromosomal segments can lead to rearrangements of the chromosome structure, such as inversions, translocations, centric split and fusion, duplications, and deletions. Chromosome rearrangement can not only promote the evolution of chromosome size, structure and shape, and composition, but also induce the chromosome number alteration [20]. Polyploidy and the subsequent return to diploidy is another important mechanism involved in chromosome evolution in plants [23]. Polyploidization can cause a dramatic increase in the chromosome number. The following diploidization undergoes complex processes, including various chromosome rearrangements, leading to chromosome evolution [21]. In fact, most of the plant species are paleopolyploidys [21]. 

## 3. Characteristics and Distribution Patterns of Repetitive Sequences in Plant Chromosomes

Repetitive sequences mainly include TEs and tandem repeats [8]. TEs, which are mobile DNA elements with the ability to move themselves independently into different parts of the genome, constitute the most dynamic and largest component of plant genomes [9,10,11,24]. According to the structural feature and the mode of transposition, TEs usually encompass two distinct classes: class I elements, also called “retrotransposons”, which propagate through an RNA intermediate; and class II elements, which transpose directly via a DNA intermediate, and are thus called “DNA transposons”. Retrotransposons can self-replicate when transposed, so are the most abundant class of plant TEs. In contrast, DNA transposons move by a direct “cut-and-paste” mechanism and are usually present in a low copy number in plant genomes [25]. 

Tandem repeats, sequences organized repeatedly in tandem arrays commonly known as satellite DNAs, are also widely distributed repetitive elements in plants. This type of repeats consists of a large number of repeat units, and usually has 50–1000 bp units [26]. The quantities of tandem repeats vary significantly in different plant species. For example, at least 19 different tandem repeat elements were observed in the *Pisum sativum* genome [27,28]. In *Raphanus sativus*, in addition to 5S rDNA and 45S rDNA, three tandemly organized repeats made up 13.93% of the genome [29]. However, we analyzed the genome of *Humulus scandens* by next-generation sequencing and subsequent satellite analysis using TAREAN [30], and did not detect remarkable tandem repeats, except for 5S and 45S rDNA [31]. 

Although the total quantitative amount of TEs is enormously variable, even between closely related plant lineages, all major representatives of TE types appear to be present in every investigated plant genome. A number of studies regarding the repetitive sequence location on chromosomes have revealed that different repeated elements have distinct distribution patterns in plant chromosomes. 

**Figure 1:**
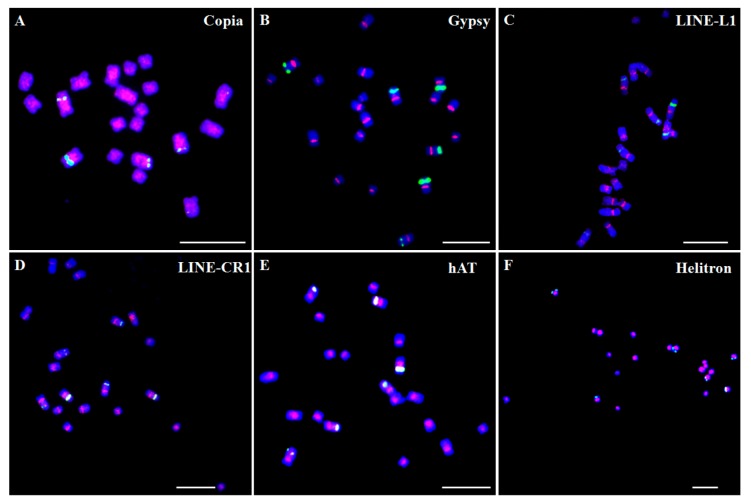
Distribution patterns of different transposable elements (TE) on chromosomes in *Asparagus officinalis*. The TE sequences were labeled with Texas Red (red signal), whereas 45S rDNA was labeled with Chroma Tide Alexa Fluor 488 (green signal), and the chromosomes were counterstained with DAPI (blue). (**A**) Ty1-copia; (**B**) Ty3-gypsy; (**C**) LINE-L1; (**D**) LINE-CR1; (**E**) hAT; (**F**) Helitron. The fluorescence in situ hybridization (FISH) images were captured with an ANDOR CCD camera under an Olympus BX63 fluorescence microscope. Bars = 10 μm.

The majority of the Ty1-*copia* family long terminal repeat (LTR) retrotransposons were generally distributed over the length of the chromosomes and were frequently found in the regions around the centromeres (pericentromere regions). Using the *copia* reverse transcriptase domain sequences as probes, fluorescence in situ hybridization (FISH) results showed that the Ty1-*copia* elements represented a dispersed distribution throughout most of the chromosomes with reduced hybridization mainly at the centromeric regions, nucleolus organizing regions, and telomere regions, such as in *Arabidopsis thaliana* and *Cicer arietinum* [32,33]. We also analyzed the chromosome distribution patterns of the main types of TEs in *Asparagus officinalis*, and found that Ty1-*copia*-like elements were dispersed over most of the chromosomes with reduced hybridization at (sub)telomeric regions, and were more inclined to distribute in clusters at the pericentromeric regions (Figure 1A). In contrast, Ty3-*gypsy* family LTR retrotransposons were mainly distributed in the centromeres. Fox example, Ty3-*gypsy* elements primarily occupied the centromeres by sequence analysis in *Arabidopsis* [34]. One Ty3-*gypsy* element was mainly distributed in the centromeres in *A. officinalis* (Figure 1B). In fact, the two well-investigated centromere-specific retrotransposons, the CRR (centromeric retrotransposon of rice) element in rice and the CRM (centromeric retrotransposon of maize) element in maize, were also Ty3-*gyspy* type elements [35]. However, certain Ty3-*gypsy* elements were also distributed in other regions except for centromeres. For instance, a Ty3-*gypsy*-like element Retand is mainly distributed in the subtelomeric heterochromatin regions of all chromosomes [36].

Other types of retrotransposons, non-LTR retrotransposons, such as LINEs (long interspersed nuclear elements), might distribute in the centromeric and/or pericentromeric regions. In *Musa acuminata*, one LINE element localized in the centromeric region of all chromosomes [37]. Similarly, one centromere-specific LINE was also detected by CHIP-Seq analysis and FISH confirmation in the sunflower [38]. In *A. officinalis*, two types of LINE, L1 and CRE, mainly occupied the centromeric and/or pericentromeric regions of all the chromosomes (Figure 1C,D).

Different types of DNA transposons exhibit a diversity of distribution patterns. In *Arabidopsis*, MULEs and CACTA elements predominate on the flanks in the centromeres and heterochromatic knobs, whereas MITE (miniature inverted-repeat transposable element) and hAT elements did not have a remarkable bias [34]. In *A. officinalis*, two types of DNA transposons, hAT and Helitron, mainly occupied the centromeric and pericentromeric regions of the chromosomes. However, the signals of Helitron were distributed more extensively and localized on most regions of the chromosomes compared to those of hAT (Figure 1E,F).

In summary, TEs appear to disperse widely in all chromosomes of the complements and show distinct patterns either in centromeres, in pericentromeres, or along all the chromosomes. In contrast, tandem repeats usually occupied specific chromosome pairs or distributed in specific regions, such as the centromeric, subtelomeric, or other easy-distinguished regions. Elements of tandem repeats that exclusively occupied one pair of chromosomes were found in several plant species, such as in the sunflower [38] and *P. sativum* [28]. In *M. acuminata*, tandem repeats CL18 and CL33 localized on one and two pairs of chromosomes, and a simultaneous hybridization analysis revealed that these two tandem repeats co-localized on one pair of chromosomes [37]. Four different tandem repeats had different distribution manners in the *Camellia japonica* genome. CajaSat1 and CajaSat2 clearly localized in the subtelomeric regions and centromeres of all chromosomes, respectively. CajaSat 3 is detectable as discrete signals on three chromosomes only, whereas CajaSat 4 showed clear enrichment in subtelomeric and pericentromeric regions [39]. rDNA as a special type of tandem repeat is mainly located on the second constraints of at least one pair of chromosomes. The 45S rDNA and 5S rDNA localized on three pairs and one pair of chromosomes, respectively, in *A. officinalis* (Figure 1).

It should be noted that sex chromosomes, which usually determine the sex type of dioecious plants and are derived from autosomes, were implemented with more repetitive sequences than autosomes. Many sex chromosome-specific repetitive sequences were identified in several dioecious plants [13]. For instance, one type of DNA transposon and both tandem repeat elements RAYSI and RAYSIII were distributed exclusively on Y_1_ and Y_2_ chromosomes, whereas RAYSII was specific for the Y1 chromosome in *Rumex acetosa* [40,41,42]. In *Carica papaya*, several repetitive sequences located on the hermaphrodite-specific region on the Y chromosome (HSY) and others were distributed in the X chromosome [43]. The highly accumulated repetitive sequences on the sex chromosomes played important roles in the sex chromosome evolution, such as heterochromatization initiation and spreading, recombination suppression, and Y chromosome degeneration [13].

In general, the distribution pattern of repetitive sequences may reflect different types of heterochromatin regions. The distribution pattern of repetitive sequences is opposite to that of the density of genes on the chromosomes. That is, repetitive sequences usually occupy the heterochromatic regions with a high TE density and low expression, whereas genes predominate in the euchromatic regions with a low TE density and high gene density and expression [44,45]. This distribution pattern is beneficial for heterochromatic TE activity inhibition and gene expression in euchromatic regions. However, some repetitive sequences are also found near or inserted into the genes. Such repetitive sequences are usually important for regulation of the nearby or inserted gene expression [46,47,48].

## 4. Functional Aspects of Repetitive Sequences in Plant Chromosome Evolution

### 4.1. Repetitive Sequences and Evolution of Chromosome Size and DNA Composition

The size of a chromosome is a conspicuous feature of a given plant species. Generally, the chromosome size of monocotyledon species is larger than that of dicotyledon plants, and the chromosome size of temperate plants is larger than that of tropical plants [49]. The total size of metaphase chromosomes is generally determined by the nuclear DNA content. For example, the total chromosomal length, as well as the total chromosomal area, in the genus *Oryza* is positively correlated with the nuclear DNA content [50]. It should be noted that the average chromosome size is not correlated well with the nuclear DNA content, because it not only depends on the amount of nuclear DNA, but is also influenced by the number of chromosomes of a complement [51].

**Figure 2:**
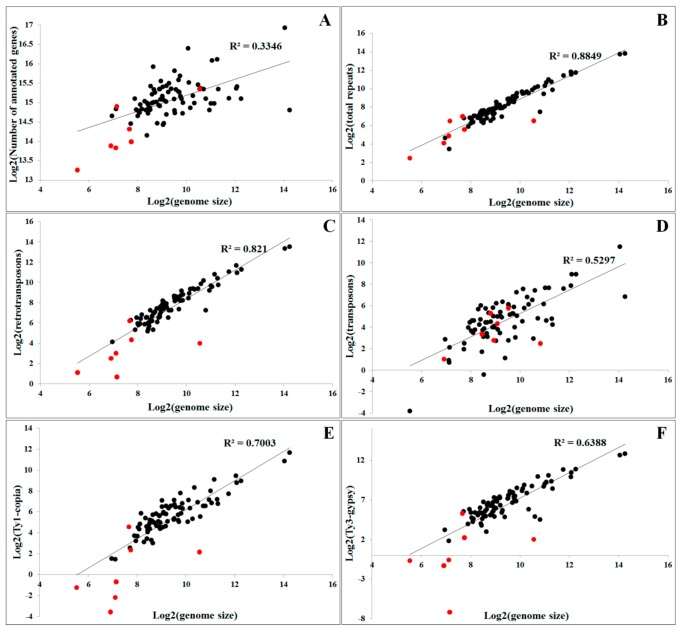
Correlation analysis between genome size and gene numbers or quantities of different types of repetitive sequences in plants. (**A**) genome size versus genes; (**B**) genome size versus repetitive sequences; (**C**) genome size versus retrotransposons; (**D**) genome size versus transposons; (**E**) genome size versus Ty1-*copia* elements; (**F**) genome size versus Ty3-*gypsy* elements. This figure is drawn based on the data presented in Table S1. The black dots represent the analysis from data of higher plants, whereas the red dots indicate the analysis from data of lower algal plants.

The main mechanisms that contribute to genome expansion in plants are polyploidization and the proliferation of repetitive DNA sequences, particularly TEs [52]. The accumulation of repetitive sequences, particularly retrotransposons, emerges as a major contributor of genome size variation [53], largely due to their intrinsic amplification ability. For example, the genome size in plants, such as *Oryza australiensis* and *Gossypium* spp, is doubled due to the retrotransposon activity [54,55]. In the genus *Asparagus*, retrotransposon proliferation is probably responsible for the larger genome size in dioecious species [56]. The genome size of the dioecious plant *Silene latifolia* is more than twice that of the gynodioecious *Silene vulgaris*, largely due to the expansion of *Ogre* retrotransposons [57]. It is now recognized that the quantity of repetitive DNA sequences is more closely correlated with the genome size than the number of coding genes [58]. We performed correlation analysis between genome size and quantities of different types of repetitive DNA or gene numbers in the plant species with sequenced genomes (Table S1). The results showed that the genome size is not correlated with gene numbers (R^2^ = 0.3346, Figure 2A). However, the genome size correlated well with the quantity of repetitive DNAs (R^2^ = 0.8849, Figure 2B). Comparisons between genome size and different classes of repetitive sequences revealed that a greater positive correlation was recorded for retrotransposons than for transposons (R^2^ = 0.821 versus R^2^ = 0.5297, Figure 2C,D). Among the retrotransposons, LTR retrotransposons showed good correlation with genome size (R^2^ = 0.774, data not shown), whereas no significant difference was observed between the contributions of Ty1-*copia* and Ty3-*gypsy* LTR retrotranposons (R^2^ value was 0.7003 and 0.6388, respectively, Figure 2E,F). These data suggest that the differential proliferation of repetitive sequences, especially retrotransposons, has largely contributed to differences in genome size observed between species.

In addition, such correlation between genome size and different types of repetitive sequences is enhanced in higher plants. If we remove the genome data of seven lower algae, the R^2^ value between genome size and various repetitive sequences, including total repetitive sequences, retrotranposons, LTR retrotransposons, Ty1-*copia*, and Ty3-*gypsy*, can be increased to 0.9062, 0.8935, 0.8683, 0.7824, and 0.7555, respectively. However, the R^2^ value between genome size and transposons was decreased from 0.5297 to 0.4552 after removing the lower plant data. The analysis indicated that retrotransposons contributed more significantly to the genome size in higher plants than in lower plants. In contrast, transposons more highly influenced the genome size in algae than in higher plants. We are not sure whether this phenomenon is due to the genome divergence between lower algae plants and higher plants, or is only due to the analyzed algae genomes having a smaller size. More data should be collected to study the impact of different classes of TEs on distinct lineages of plants.

The total chromosome size is also highly positively correlated with the repetitive sequence contents because total chromosome size is mainly determined by the nuclear DNA content. Numerous studies within different lineages confirmed this general trend. Among the 11 species of the genus *Oryza*, *O. australiensis*, with the largest genome and longest chromosome size, showed the overall amplification of genome-specific DNA sequences throughout the chromosomes, whereas *Oryza brachyantha*, with the smallest genome and shortest chromosome size, had limited repetitive sequences [50].

The most direct evidence is the size of sex chromosomes of dioecious plants. A number of dioecious plants have a larger Y chromosome than X chromosome, such as in *S. latifolia* [59], *Coccinia grandis* [60], and *Cannabis sativa* [61]. Evidence has suggested that the larger Y chromosome was formed by the accumulation of a large number of repetitive sequences. For example, the Y chromosome in *S. latifolia* is the largest chromosome in male metaphase and it has accumulated a large number of repetitive DNAs. TE insertions are presented at more highly predicted frequencies at sites on the Y chromosome than on the other chromosomes by transposon display analysis [59]. Additionally, in *C. grandis*, the large Y chromosome is mainly due to the accumulation of various repetitive sequences, such as Ty1-*copia* and Ty3-*gypsy* elements, unclassified elements, and tandem repeats [60].

Although repetitive sequences are major contributors to plant genome size, the prevalence of particular repeat families differs dramatically among different plant groups. In many cases, a limited number of repetitive types are highly amplified in one lineage. For example, a single Ty3-*gypsy*-like retrotransposon accounts for approximately 38% of the genome of *Vicia pannonica* [62], and the accumulation of a single-type LTR retrotransposon that belongs to the Del subgroup plays vital roles in the *Capsicum annuum* genome expansion [63]. In several cases, the amplification of a specific family is observed in several related species [64], but the copy number normally showed a large difference in close relatives [65]. In other cases, a number of genomes comprised several TE families with similar quantities. However, many individual TE families in several species were amplified causing genome expansion, such as in *Picea abies*, for which more than 86% of the repetitive elements recovered were singletons [24]. These observations reveal that the accumulation pattern of repetitive sequences not only depends on the element itself, but also on the genome. Several repetitive elements can escape the control in a particular genome, and certain genomes are more tolerant of the amplification of repeats [66].

In addition, the accumulation of repetitive sequences can obviously influence the DNA composition of chromosomes. For example, 79.2% of the male-specific regions on the Y chromosome (MSY) and 67.2% in the X chromosome counterpart are occupied by repetitive sequences, whereas the ration of repetitive sequences in the entire genome is 51% [67]. Furthermore, the repetitive sequences are more tolerant of mutations, mainly because they are less influenced by the selective pressure [68,69]. Thus, the amplification, deletion, and mutation of the repetitive sequences contributed largely to the DNA composition of chromosomes during the evolution process.

### 4.2. Repetitive Sequences and Evolution of Chromosome Structure and Shape

The structure and shape of chromosomes can be altered by chromosome rearrangement, including insertion, duplication, deletion, centric split and fusion, inversion, and translocation. Comparative cytogenetic studies revealed extensive chromosome rearrangements in many plant species, such as in the Brassicaceae family [70,71,72], Solanaceae family [19,73], and grass family [74,75]. For example, the difference between *Arabidopsis lyrata* and *A. thaliana* was mainly explained by 10 major rearrangement events, including five inversions, two translocations, and three fusion/fissions [71]. The differences in the structure, shape, and numbers of chromosomes in related species, both in animals and plants, are due to the syntenic blocks being assembled in different combinations. Blocks that are fused together in one species can be separated on different chromosomes in another. Segments within blocks can be duplicated, lost, or inverted [76,77,78]. The current karyotype of a given species is formed by complex chromosomal rearrangement usually combining two or more rearranging events, and the process is still going on. For example, BAC-FISH analysis showed that the genomes of *Brachypodium distachyon*, *Brachypodium sylvaticum*, and *Brachypodium pinnatum* were differentiated by chromosomal rearrangements, such as duplications, translocations, and inversions. For instance, the presence of a chromosome pair carrying an additional site for Bd2/1 in *B. pinnatum* might have resulted either from a duplication or a translocation event, and the Bd2/11 in *B. sylvaticum* was possibly formed by a reciprocal translocation between the chromosome carrying sites for Bd2/10 and Bd2/11 [79].

Increasing evidence suggests that major structural chromosomal repatterning is frequently associated with cytogenetically detectable heterochromatic regions composed of repetitive DNA sequences [70,80,81,82]. Repetitive sequences, especially TEs, are involved in various chromosomal rearrangements. Early in 1946, Barbara McClintock suggested that transposons can cause chromosome breakage and dissociate the acentric fragment from the rest of the chromatid [83]. Cytogenetic evidence showed that the *En/Spm* transposons were involved in the ongoing chromosomal rearrangement leading to the rise of a new fertile plant population of *Aegilops speltoides* [84].

**Figure 3:**
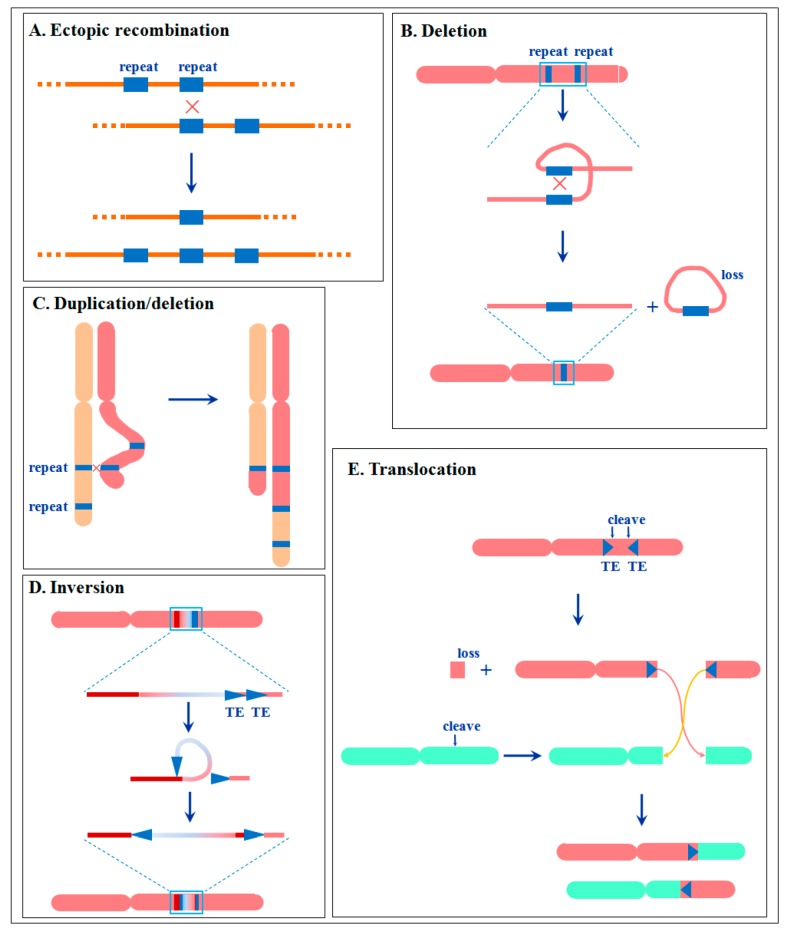
A simple scheme summarizing the effect of repetitive sequences involved in individual processes on chromosome structure. See details in text.

Various factors can cause a double-strand break (DSB) on a chromosome, and the chromosomal rearrangements may be the result of illegitimate recombination during the process of DSB repair, either via the direct joining of ends between different DSBs or through recombination with ectopic homologous sequences (Figure 3A). The ectopic recombination usually occurred at ectopic homologous sequences as a template for recombination repair. Thus, the repetitive sequences provide the ideal target region. It has been observed that the primary rearrangements are nearly exclusively located in heterochromatic regions enriched in similar highly repetitive DNA sequences [70,85].

Ectopic recombination between homologous repetitive sequences within one chromosome can cause a shorter chromosome and a chromosome fragment, followed by the loss of the chromosome fragment (Figure 3B). In fact, the small Y chromosomes in many animals and several dioecious plants are probably formed by this mechanism [86]. Ectopic recombination between homologous repetitive sequences between homologous chromosomes leads to the duplication of one chromosome and the deletion of another (Figure 3C). In cucurbit species, the gain/loss-associated centromere reposition of pericentromeric heterochromatin sequences caused distinct alteration of the structure and shape of derived chromosomes between cucumber and melon. For example, the cucumber chromosome 6 is a metacentric chromosome with little heterochromatin, whereas the related melon chromosome 1 is a subtelocentric chromosome with a large amount of heterochromatin in the pericentromeric regions [87]. 

Other chromosomal rearrangements, such as inversion, translocation, and centric fusion and fission are also related to repetitive sequences. For example, various types of repetitive sequences may play a role by facilitating the formation of secondary structure intermediates between the single-stranded DNA ends that recombine during chromosome rearrangements, such as translocations, and gross deletions in humans [80]. In *Drosophila buzzatii*, the commonly occurring polymorphic inversions were probably formed by ectopic recombination, during which the breakpoints contain large insertions corresponding to transposable elements [88]. Apparently, these TEs contributed to natural inversions in *D. buzzatii*. Sequence analysis suggested that one 1.17 Mb inversion between Col-0 and Ler *Arabidopsis* was caused by the activity of a transposon Vandal5, which is a Mutator-like (Mule) transposon. According to the arrangement of the sequences at the distal and proximal breakpoints of the inversion, it is inferred that the 5′-end of the Vandal5 transposon inserted into the third exon of an F-box protein-coding gene, whereas the other end of the transposon remained attached to the original donor site. Recombining the two free ends resulted in the inversion (Figure 3D) [72]. 

Furthermore, translocations are also related to repetitive sequences, and ectopic recombination between homologous repetitive sequences between different chromosomes leads to the reciprocal translocation events. The Ty retrotransposon elements driving translocation events, and other duplication and deletion events, account for the chromosome length polymorphism of enological stains of *Saccharomyces cerevisiae* [89]. Molecular and cytogenetic analyses showed that 19 major chromosomal rearrangements, including 17 reciprocal translocations and two large inversions, were detected in the analyzed maize lines. The junctions of all these 19 chromosome rearrangements contained Ac termini and eight bp target site duplications. The results strongly indicated that excision of the Ac and fAc (a fractured Ac element) termini followed by insertion at a chromosomal target site leads to a rearrangement of the sequences flanking the transposon termini. After the cleavage of Ac and fAc ends by Ac transposase, the Ac/fAc termini inserted into a site on the opposite arm of the same sister chromatid could generate a pericentric inversion, whereas the transposon ends inserted into a site in another chromosome could produce a reciprocal translocation (Figure 3E) [90]. It should be noted that the mechanism of repetitive sequences involved in duplication and deletion is presently well established, as shown in Figure 3B,C. However, the roles played by repetitive sequences in inversion and translocation have not yet been clearly understood. Figure 3D,E only show partial possible mechanisms, and a comprehensive view of the relationship between repetitive sequences and inversion/translocation still needs more evidence.

### 4.3. Repetitive Sequences and Evolution of Chromosome Number

Chromosome numbers can be altered by ploidy mutations involving the entire complement (polyploidy) or individual chromosomes (aneuploidy) [20]. In addition, chromosome rearrangements, such as chromosome fission or fusion, can also increase or decrease the number of chromosomes [70,91]. In general, the base chromosome number reduction in monodicots is usually caused by nested chromosome fusions, whereas in eudicots, end-to-end fusions are mostly involved [20,70,79]. Nested chromosome fusion is a process during which a whole chromosome is inserted by its telomeres into a break in the centromeric region of another chromosome [91]. For example, comparative cytogenomics analysis among *Brachypodium*, sorghum, rice, and wheat revealed that the current five *Brachypodium* chromosomes were formed from a five-chromosome ancestral genome via a 12-chromosome intermediate involving seven major chromosome fusions caused by nested chromosome insertions [77]. While in eudicots, end-to-end fusions played an important role in base chromosome number reduction. For example, the karyotypes in *A. thaliana* (*n* = 5) and of related species with six or seven chromosome pairs were derived from an ancestral karyotype with eight chromosome pairs. Chromosome fusions in *A. thaliana* resulted from the generation of acrocentric chromosomes by pericentric inversions, reciprocal translocation between two chromosomes (one or both acrocentric), and elimination of a minichromosome that arose in addition to the fusion chromosome [70]. In addition, centric fission can cause an increase in the base chromosome number and karyotype symmetry. For example, comparative linkage mapping analysis showed that the genomes of closely related species, *Mimulus lewisii* and *Mimulus guttatus*, present strong segmental synteny, and compared to the ancestral base number 8 of *M. lewisii*, the reconstruction of 14 *M. guttatus* chromosomes requires at least eight fission events plus two fusion events [92].

As described above, the chromosome arrangement frequently occurred at the chromosome regions replete with repetitive sequences. Thus, the chromosome number change associated with chromosome arrangement leading to chromosome number alteration is often related to repetitive sequences. In fact, in the process of nested chromosome fusions that mostly occur in grasses, the concerned centromeric region and telomeric region are embedded with an abundance of repetitive sequences [77,93]. In has also been reported that repetitive sequence-abundant regions, such as constitutive heterochromatin, GC-rich DNA, and rDNA are implicated in chromosomal rearrangements when the basic chromosome number descends in the *Reichardia* genus [82].

Overall, rapid chromosomal evolution is driven by the activity of repetitive sequences. Although the exact mechanism of repetitive sequences involved in chromosomal evolution remains largely an enigma, it is speculated that repeated sequences within heterochromatin may affect karyotypic evolution by facilitating rearrangements that have a minimal deleterious impact on the genome [5]. Repetitive sequences, especially TEs, can amplify themselves; can stimulate chromosome rearrangement, including the inversion, duplication, or deletion of adjacent DNA, translocation, chromosome breaking and repairing, and aborted transposition; or can cause ectopic recombination between homologous repeated elements at different chromosomal locations. Therefore, the genome structure of a species is largely the outcome of TE actions and of the cellular processes that act on TEs [94].

## 5. Epigenetic Modification and Plant Chromosome Evolution

Although TEs and other repetitive sequences are extensively distributed in plant genomes and play essential roles in chromosome evolution, the majority of the TEs are dormant or inactivated, and only a tiny fraction of TEs are transpositionly active [95]. This is mainly achieved by epigenetic silencing evolved to control the proliferation of TEs and eliminate their perceived damage effect [69]. Epigenetic modification, including DNA methylation, histone modification, and small RNA pathway regulation, represents a sophisticated mechanism used to ameliorate the deleterious effects of TEs and other types of intrusive DNA, such as transgenes [13,96,97]. TEs and the derived repetitive DNA sequences are preferred targets of the epigenetic modification [98,99]. The hypomethylation level induced by a demethylation reagent could cause the activation and transposition of TEs in a fungus [100]. Epigenetic modification affects most aspects of plant growth, development, and reproduction processes [101,102,103]. We hypothesize that the epigenetic modification, intertwined with TEs, also plays important roles in chromosome evolution in plants.

### 5.1. Epigenetic Modification and Heterochromatin

Eukaryotic genomes often contain two distinct parts, heterochromatin and euchromatin. Heterochromatin is normally concentrated in the centromeric, pericentromeric, and telomeric regions, NORs, and some distinct knobs [104,105]. In contrast to euchromatin, heterochromatin is generally characterized by repetitive sequence accumulation, post-translational histone modifications, and epigenetic gene silencing [104,106]. In *A. thaliana*, DNA methyl transferase, H3K9 histone methylase, and histone deacetylase (H4K16) are involved in heterochromatin assembly [107,108].

Heterochromatin is essential for normal chromosome organization, centromere function, and telomere protection [109,110,111]. The epigenetic modification is frequently involved in the condensed heterochromatic regions [112]. Such epigenetic silencing through the formation of heterochromatin, which involves DNA and histone methylation and RNA interference (RNAi) pathways, has been shown for preventing the proliferation of TEs and suppressing the unfavorable transcription of genes [113,114]. It has been revealed that the mRNAs produced by TEs were degraded by the RNAi pathway, and the TE activity was thereby reduced [115]. In addition, the RNAi pathway induces the transcriptional silencing of TEs by recruiting chromatin-modifying elements to transposable element loci for modification of the targeted region of chromatin. Modifications in the targeted TE region, combined with other complex processes, including histone modifications and other unknown mechanisms, may cause heterochromatin formation [13,116]. In *Arabidopsis*, small interfering RNAs correspond to TEs, and related repeated sequences can guide the chromatin remodelling of ATPase DDM1 (Decrease in DNA Methylation 1) for heterochromatin formation [96,108]. Furthermore, the heterochromatin may recruit many chromatin-modifying elements, such as histone methylatransferase, DNA methyltransferase, and other chromatin-modifying elements, thereby causing the expansion of heterochromatin to the adjacent regions [13]. For example, TE-triggered DNA methylation and H3K9me2 can spread to the nearby regions, thereby influencing the chromatin state of nearby sequences in maize [117,118].

### 5.2. Epigenetic Modification and Centromere Function

Epigenetic modification is essential for centromere function. Centromeres are specialized chromosomal structures that are important for spindle attachment and chromosome separation, which have now been shown to incorporate various classes of repetitive sequences that are embedded in heterochromatic regions [119]. Despite the evolutionarily conserved function, centromeric DNA sequences are dramatically variable, even among closely related species [120,121]. It is suggested that centromere function is independent of its underlying DNA sequences but governed by epigenetic mechanisms, which is still poorly understood [122,123]. DNA methylation and histone modifications are potential epigenetic marks for centromere identity and function [124].

The functional centromere can be inactivated easily, and the inactivation is associated with epigenetic change [125]. For example, the DNA sequences in the normal B chromosome centromere exhibit hypomethylation, whereas the inactivation of this centromere is accompanied by an increased methylation level [126]. In wheat, a chromosome was reported to have three centromeres, one large and two small. The small centromeres were always inactivated, and the inactivation was also associated with enhanced H3K27me2 (dimethylation of lysine 27 on histone H3) and H3K27me3 (trimethylation of lysine 27 on histone H3) in the related pericentromeric chromatin [123]. Furthermore, if normal centromeres are lost or inactivated, regions without centromeric repeats can recruit CENH3 (centromere histone H3), a conventional histone H3 in plants, and other centromere-associated proteins to assemble a new functional centromere [127,128]. In maize, a new functional centromere could be formed after the loss of centromeric sequences and gain of ectopic sequences, which suggested that functional centromeres may be formed without the known centromere-specific sequences. However, the maintenance of a high DNA methylation level appears to be crucial for the proper function of a new centromere [128]. In maize inbreds, the establishment of neocentromeres at novel sites was accompanied by centromeric repeat (centC) deletions at the old centromere and new repetitive sequence accumulation, which can recruit sequence-specific binding proteins that are favorable for establishing the necessary epigenetic environment of the novel [129].

### 5.3. Epigenetic Regulated Transposable Elements Reactivation and Chromosome Evolution

Under normal conditions, the majority of TEs and the derived repetitive sequences are dormant and inactive, mainly due to the epigenetic modification status. However, when the genome homeostasis is disturbed, such as biotic and abiotic stresses, hybridization, tissue culture, and polyploidy, the TEs can be released from suppression and thereby become activated [130,131,132]. Such reactivation is caused by the alteration of epigenetic modification and can result in considerable effects on the genome and chromosome structures. The interspecific hybrids of *Macropus eugenii* and *Wallabia bicolor* have clearly longer centromeres, which are caused by undermethylation-associated retroelement amplification in the centromere regions [133]. The DNA methylation level was lower in the hybrids of *Chrysanthemum morifolium* and *Leucanthemum paludosum* than in the parental lines [134]. In Helianthus, inter-species hybrids have dramatically larger genomes [135] and chromosomal rearrangements [136], and such alteration is associated with TE reactivation and amplification [137,138]. These results indicate that the suppression of DNA methylation and subsequent TE reactivity in hybrids could facilitate rapid karyotypic evolution. It is surprising that an *Ogre* family is nearly absent on the Y chromosome but is ubiquitous in the genome of *S. latifolia*. Further combined analysis suggested that 24-nucleotide small RNA might drive the CHH methylation of *Ogre* LTRs, and such small RNA-mediated silencing might cause the absence of the *Ogre* family on the Y chromosome [139]. In synthetic and natural allopolyploids, the genomes frequently underwent rapid epigenetic changes, including alteration in cytosine methylation manners, the relaxation of imprinting genes, the silencing and activation of homologous genes, and the reactivation of dormant TEs [140]. These epigenetic changes are inter-related and may promote gene diversification, contribute to genetic and cytological diploidization, and facilitate intergenomic coordination, thus bearing direct relevance to polyploidy evolution, adaptation, and speciation [140,141].

## 6. Conclusions and Perspectives

Repetitive sequences and related epigenetic modification must play at least some fundamentally important functional roles in chromosome evolution. The characterization and pattern of repetitive DNA sequences are vital to understanding the dynamics and mechanism of chromosomal evolution among plant genomes, even in eukaryotic genomes. However, at this point in our knowledge, the mechanism of repetitive sequences and epigenetic modification contributing to chromosome evolution is still poorly understood. For example, chromosome rearrangements frequently occur at repetitive regions, but how repetitive sequences promote chromosome rearrangements is not clearly established. Furthermore, the mechanism of epigenetic modification involved in chromosome evolution is only preliminary, and occasionally, the results are controversial. Thus far more detailed evidence in various species should be obtained to reach further general conclusions about the functional roles of repetitive sequences and epigenetic modification in plant chromosome evolution. The combination of the current efficient high-throughput sequencing and high-resolution cytogenetic technique will facilitate this research. In addition, the recent emergent repetitive sequence analysis tools, such as RepeatExplorer [142] and TAREAN [30], could facilitate the function analysis of repetitive sequences in chromosome evolution. We expect that repetitive sequences will be increasingly appreciated for their dynamic contributions to the evolutionary process of plant chromosomes.

## Supplementary Materials

The following are available online at www.mdpi.com/2073-4425/8/10/290/s1. Table S1: Information on genome size, gene numbers, proportion, and quantities of different types of repetitive sequences in plants. All the sequenced plant genomes were collected, whereas only species with complete information about genes and repetitive sequences were displayed.
